# Preoperative Low Serum Bicarbonate Levels Predict Acute Kidney Injury After Cardiac Surgery

**DOI:** 10.1097/MD.0000000000003216

**Published:** 2016-04-01

**Authors:** Su-Young Jung, Jung Tak Park, Young Eun Kwon, Hyung Woo Kim, Geun Woo Ryu, Sul A. Lee, Seohyun Park, Jong Hyun Jhee, Hyung Jung Oh, Seung Hyeok Han, Tae-Hyun Yoo, Shin-Wook Kang

**Affiliations:** From the Department of Internal Medicine, College of Medicine, Severance Biomedical Science Institute, Brain Korea 21 PLUS, Yonsei University, Seoul, South Korea.

## Abstract

Acute kidney injury (AKI) after cardiac surgery is a common and serious complication. Although lower than normal serum bicarbonate levels are known to be associated with consecutive renal function deterioration in patients with chronic kidney injury, it is not well-known whether preoperative low serum bicarbonate levels are associated with the development of AKI in patients who undergo cardiac surgery. Therefore, the clinical implication of preoperative serum bicarbonate levels on AKI occurrence after cardiac surgery was investigated. Patients who underwent coronary artery bypass or valve surgery at Yonsei University Health System from January 2013 to December 2014 were enrolled. The patients were divided into 3 groups based on preoperative serum bicarbonate levels, which represented group 1 (below normal levels) <23 mEq/L; group 2 (normal levels) 23 to 24 mEq/L; and group 3 (elevated levels) >24 mEq/L. The primary outcome was the predicated incidence of AKI 48 hours after cardiac surgery. AKI was defined according to Acute Kidney Injury Network criteria. Among 875 patients, 228 (26.1%) developed AKI within 48 hours after cardiac surgery. The incidence of AKI was higher in group 1 (40.9%) than in group 2 (26.5%) and group 3 (19.5%) (*P* < 0.001). In addition, the duration of postoperative stay in a hospital intensive care unit (ICU) was longer for AKI patients and for those in the low-preoperative-serum-bicarbonate-level groups. A multivariate logistic regression analysis showed that low preoperative serum bicarbonate levels were significantly associated with AKI even after adjustment for age, sex, hypertension, diabetes mellitus, operation type, preoperative hemoglobin, and estimated glomerular filtration rate. In conclusion, low serum bicarbonate levels were associated with higher incidence of AKI and prolonged ICU stay. Further studies are needed to clarify whether strict correction of bicarbonate levels close to normal limits may have a protective role in preventing further AKI development.

## INTRODUCTION

Acute kidney injury (AKI) is a common postoperative complication in patients undergoing cardiac surgery. The incidence rates of cardiac surgery-associated AKI (CSA-AKI) have been reported to be 7.7% to 40% depending on patient populations,^1–5^ and CSA-AKI significantly increases mortality risk.^3,6,7^ In addition, patients who develop CSA-AKI frequently require renal replacement therapy (RRT), which lengthens ICU stay and worsens long-term morbidity.^3^ Mild deterioration in renal function after cardiac surgery not requiring RRT is also significantly associated with poor clinical outcome.^8^

Several factors have been found responsible for the development of CSA-AKI, including generation of reactive oxygen species, inflammatory cytokines, and ischemia-reperfusion injury.^9–11^ Even though the pathogenesis of CSA-AKI is not fully understood, it seems to be a multifactorial interaction between hemodynamic, inflammatory, and direct nephrotoxic injuries to renal cells.

Acidosis has been considered to be implicated in the pathogenesis of renal injury. It could aggravate tubular damage by increasing tubular ammonia production, which activates the complement system and leads to tubulointerstitial injury.^12,13^ Moreover, activation of the intrarenal renin–angiotensin system was observed in mice with acid overload.^14^ Furthermore, several observational studies have shown a clear relationship between metabolic acidosis and rapid decline in renal function in patients with early or advanced chronic kidney disease (CKD).^15–19^ There was also a significant association of acidosis with all-cause mortality in patients with CKD.^20–22^ However, the impact of serum bicarbonate levels on the development of CSA-AKI has not yet been fully elucidated. In this study, we aimed to explore the clinical implication of preoperative serum bicarbonate levels in the development of AKI after cardiac surgery.

## MATERIALS AND METHODS

### Patient Selection and Outcome

Data were retrieved based on the medical records of 994 patients who underwent coronary artery bypass graft (CABG) or valve surgery at Yonsei University Health System in Seoul from January 2013 to December 2014. Patients were excluded if they met the following criteria: <18 years of age, history of hemodialysis or peritoneal dialysis before surgery, and incomplete data. Thus, a total of 875 patients were included in the final analysis. The study protocol was approved by the Institutional Review Board of Yonsei University Health System (Approval number 4-2015-1020). Because the present study was a retrospective, medical-record-based study and the study subjects were de-identified, the board waived the need for written consent from the patients.

### Laboratory and Clinical Data

Demographic and clinical data at the time of surgery—including age, sex, comorbidities, and type of operation—were recorded. Total serum carbon dioxide levels, which are generally used as indirect measures of serum bicarbonate concentrations, were determined by an electrode-based method (UniCel DxC 800; Beckman Coulter, Inc, Brea, CA). Laboratory data measured within 96 hours before operation were adopted for preoperative values. The following biochemical laboratory test results were also collected: hemoglobin, serum creatinine, glucose, total cholesterol, and albumin levels. The estimated glomerular filtration rate (eGFR) was calculated using the 4-variable Modification of Diet in Renal Disease Study equation.^23^

### Follow-Up and Endpoints

All patients were followed up until hospital discharge after operation. The patients were divided into 3 groups based on preoperative serum bicarbonate levels, which represented group 1 (below normal levels) <23 mEq/L; group 2 (normal levels) 23 to 24 mEq/L; and group 3 (elevated levels) >24 mEq/L. The primary outcome was the development of AKI within 48 hours after cardiac surgery. AKI was defined according to Acute Kidney Injury Network (AKIN) criteria: stage 1, an increase in serum creatinine ≥26.5 μmol/L (≥0.3 mg/dL) or increase to ≥150% and 199% (1.5- to 1.9-fold) from baseline; stage 2, an increase in serum creatinine to 200% to 299% (>2- to 2.9-fold) from baseline; and stage 3, an increase in serum creatinine to ≥300% (≥3-fold) from baseline or serum creatinine 354 μmol/L (≥4.0 mg/dL) with an acute rise of at least 44 μmol/L (0.5 mg/dL) or initiation of RRT.^24^ All patients who met the AKIN criteria for stages 1, 2, and 3 were considered as having AKI. Secondary outcomes were duration of postoperative ICU stay, total hospital stay, and in-hospital mortality.

### Statistical Analysis

All statistical analyses were performed using SPSS for Windows version 20.0 (SPSS Inc, Chicago). Continuous variables were expressed as mean ± standard deviation, and categorical variables as absolute number with percentages. Comparisons between the groups were made by way of analysis of variance or Student's *t*-test for continuous variables and by the chi-squaretest or Fisher's exact test for categorical variables. The Kolmogorov–Smirnov test was performed to determine the normality of the distribution of parameters. If the resulting data did not show a normal distribution, geometric mean ± standard deviation was reported; the Mann–Whitney *U* test or Kruskal–Wallis test was used for multiple comparisons. Multivariate logistic regression analysis was performed to identify independent predictors of CSA-AKI. *P* values <0.05 were considered statistically significant.

## RESULTS

### Patient Characteristics

Baseline clinical characteristics and laboratory findings are shown in Table 1. The mean age was 60.5 years, and 518 patients (59.2%) were men. Among 875 patients, 155 (17.7%) had diabetes and 294 (33.6%) underwent CABG. Of the patients who underwent CABG, 89.5% underwent off-pump bypass surgery. Twenty-four (2.7%) patients received simultaneous CABG and valve operations. The mean preoperative serum bicarbonate level was 24.5 ± 2.9 mEq/L, and the mean eGFR before operation was 83.6 ± 23.2 mL/min/1.73 m^2^.

**TABLE 1:**
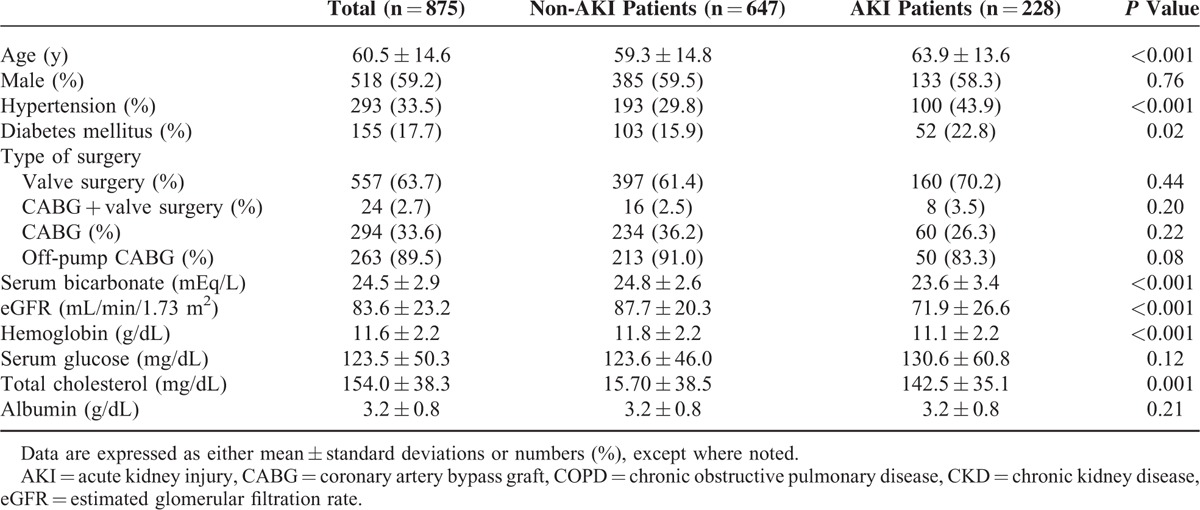
Baseline Characteristics of AKI Patients and Non-AKI Patients

### Outcomes

AKI was observed in 228 patients (26.1%) after operation. Of patients diagnosed with CSA-AKI, RRT was required in 27 (3.1%). Operation-related death occurred in 29 patients (3.3%). The mean duration of postoperative ICU stay was 3.6 days, and the mean total hospital stay duration was 19.4 days.

### Comparison Between Patients With AKI and Patients Without AKI

Compared with the non-AKI group, both mean age (63.9 ± 13.6 vs 59.3 ± 14.8 years, *P* < 0.001) and proportion of patients with hypertension (43.9 vs 29.8%, *P* < 0.001) were significantly higher in patients with CSA-AKI. Diabetes mellitus was also significantly more prevalent in the AKI group (22.8 vs 15.9%, *P* = 0.02). In addition, the proportion of patients who underwent valve replacement operation alone was significantly higher, whereas the proportion of patients who received either CABG alone or simultaneous valve replacement and CABG was significantly lower in the AKI group (*P* = 0.02). Laboratory test results revealed that serum bicarbonate levels (23.6 ± 3.4 vs 24.8 ± 2.6 mEq/L, *P* < 0.001), eGFR (71.9 ± 26.6 vs 87.7 ± 20.3 mL/min/1.73 m^2^, *P* < 0.001), and hemoglobin (11.1 ± 2.2 vs 11.8 ± 2.2 g/dL, *P* < 0.001) and serum total cholesterol concentrations (142.5 ± 35.1 vs 157.0 ± 38.5 mg/dL, *P* < 0.001) were significantly lower in patients with AKI. However, serum glucose and albumin levels were comparable between the 2 groups (Table 1).

Regarding clinical outcome, the mean durations of not only postoperative ICU stay (6.2 ± 8.5 vs 2.7 ± 2.2 days, *P* < 0.001) but also total hospital stay (28.9 ± 26.5 vs 16.0 ± 14.7 days, *P* < 0.001) were significantly longer in patients with CSA-AKI. Moreover, a significantly higher number of deaths (8.8 vs 1.4%, *P* < 0.001) was observed in the AKI group (Table 2).

**TABLE 2:**
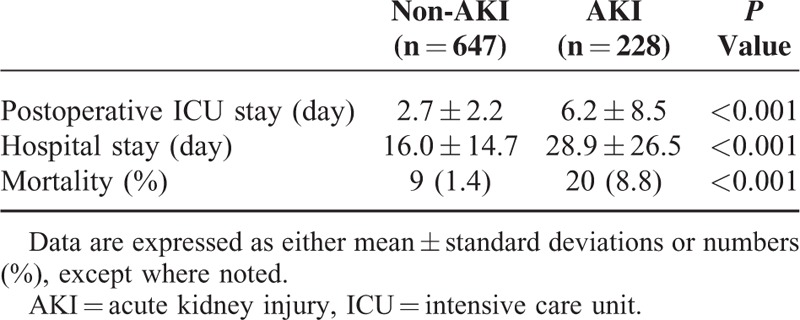
Comparison of Postoperative ICU Stay, Hospital Stay, and Mortality for AKI Patients and Non-AKI Patients

### Effect of Preoperative Serum Bicarbonate Levels on Clinical Outcome

To investigate the effect of serum bicarbonate concentrations on clinical outcome, patients were divided into 3 groups according to preoperative serum bicarbonate levels: group 1 (below normal levels) < 23 mEq/L; group 2 (normal levels) 23 to 24 mEq/L; and group 3 (elevated levels) >24 mEq/L. The groups consisted of 193, 226, and 456 patients, respectively.

**FIGURE 1:**
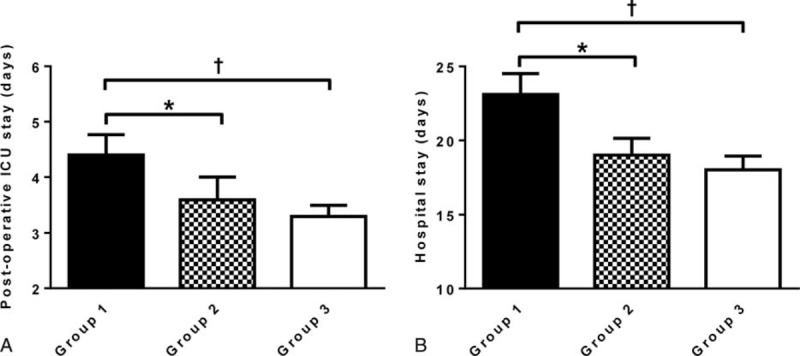
Duration of postoperative ICU and hospital stay. Comparison of (A) postoperative ICU stay duration and (B) hospital stay duration among preoperative serum bicarbonate level groups. Mean ± standard error. ∗*P* < 0.05. †*P* < 0.01. AKI = acute kidney injury, ICU = intensive care unit.

**FIGURE 2:**
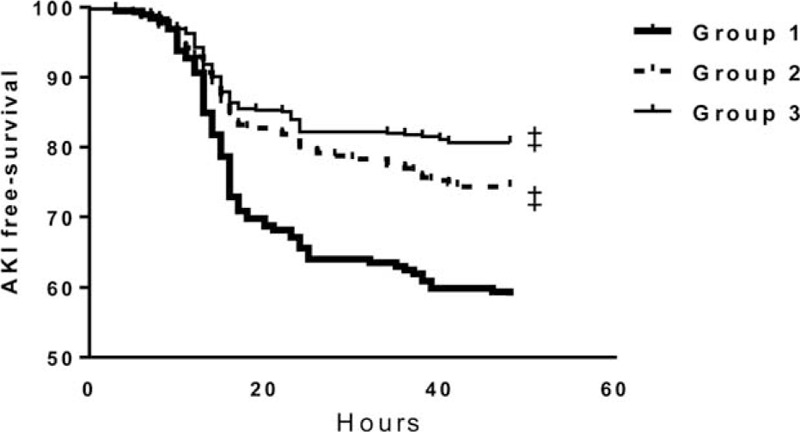
Kaplan–Meier plots for CSA-AKI according to preoperative serum bicarbonate levels. The log-rank test revealed that the occurrence rate of CSA-AKI was significantly higher in group 1 compared to groups 2 and 3. ‡; *P* < 0.001 vs group 1. AKI = acute kidney injury, CSA-AKI = cardiac surgery-associated AKI.

CSA-AKI developed in 40.9% of patients in group 1, 26.5% in group 2, and 19.5% in group 3. The incidences of AKIN criteria stages 1 and 2 were comparable among the groups, but stage 3 AKI was more common in group 1 compared to groups 2 and 3 (*P* = 0.02). AKI events were observed more significantly in group 1 compared with groups 2 and 3 (*P* = 0.001 and *P* < 0.001, respectively) (Table 3). Compared with group 2 (3.61 ± 4.3 days) and group 3 (3.30 ± 4.3 days), furthermore, the duration of postoperative ICU stay was significantly longer for patients in group 1 (4.42 ± 5.2 days) (*P* = 0.001 and *P* < 0.001, respectively) (Figure 1A). Total hospital admission duration was also significantly longer for patients in group 1 (*P* < 0.001) (Figure 1B). Kaplan–Meier cumulative survival analysis revealed that the occurrence rate of CSA-AKI was significantly higher in group 1 compared to groups 2 and 3 (*P* < 0.001) (Figure 2).

**TABLE 3:**
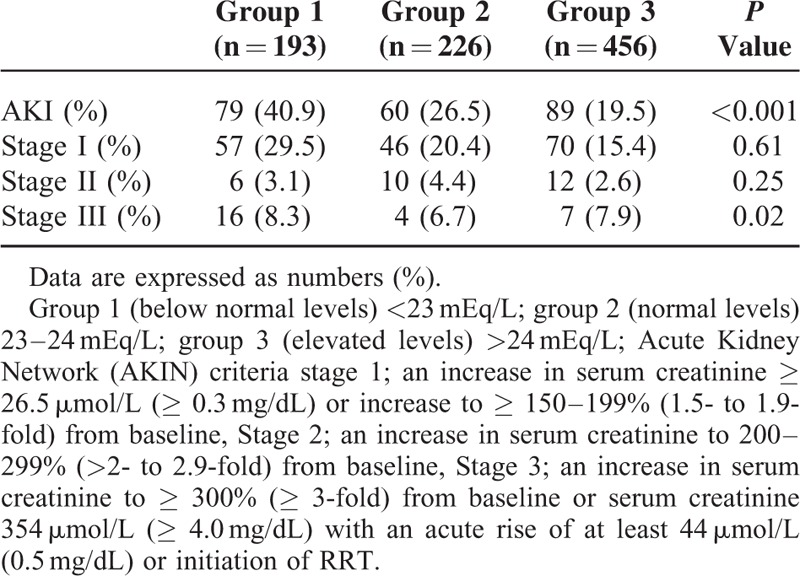
AKI Stage and Serum Bicarbonate Levels Group

### Predictors of AKI

Potential risk factors for CSA-AKI were first determined by univariate logistic regression analysis. Patients in group 1 (OR 2.86, 95% CI 1.98–4.13, *P* < 0.001) and group 2 (OR 1.49, 95% CI 1.02–2.17, *P* = 0.04) were demonstrated to have significantly higher risk of developing AKI compared with group 3 patients, suggesting that low preoperative serum bicarbonate concentrations increased the risk of AKI after cardiac surgery. Older age (OR 1.02, 95% CI 1.01–1.04, *P* < 0.001), hypertension (OR 1.84, 95% CI 1.35–2.51, *P* < 0.001), and diabetes (OR 1.56, 95% CI 1.07–2.27, *P* = 0.02) were also significantly associated with the development of AKI. Moreover, valve replacement surgery increased the risk of postoperative AKI relative to CABG (OR 1.57, 95% CI 1.12–2.20, *P* = 0.01). In contrast, increases in the hemoglobin level (OR 0.86, 95% CI 0.80–0.93, *P* < 0.001) and high eGFR (OR 0.97, 95% CI 0.96–0.98, *P* < 0.001) were found to reduce the risk of development of AKI (Table 4).

**TABLE 4:**
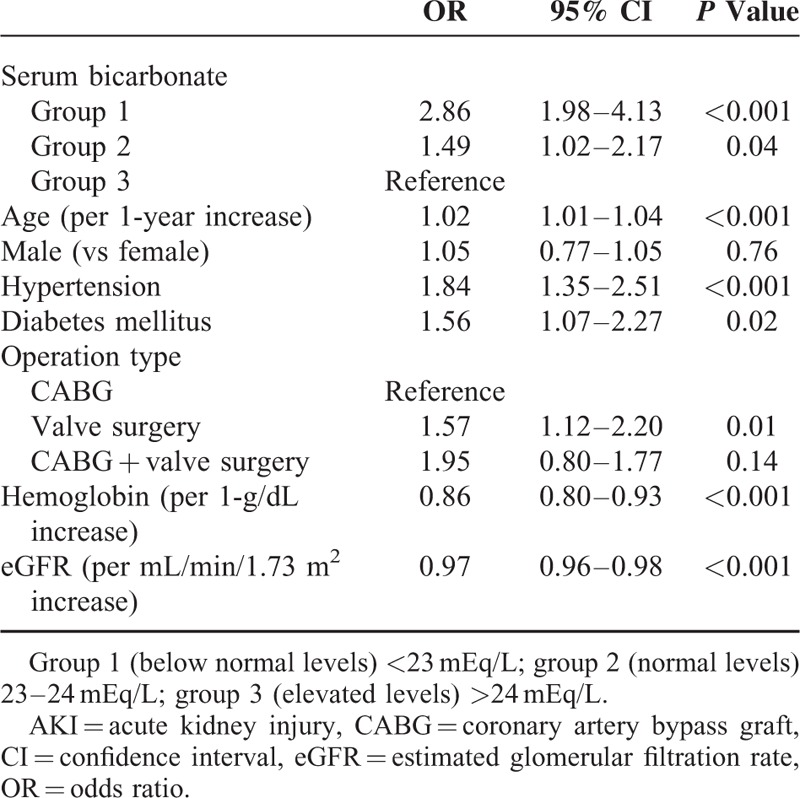
Univariate Logistic Regression Analysis of Cardiac Surgery-Associated AKI Development With Clinical and Biochemical Variables

Multivariate logistic regression analysis revealed that patients in group 1 (OR 2.36, 95% CI 1.57–3.54, *P* < 0.001) and group 2 (OR 1.54, 95% CI 1.03–2.26, *P* = 0.03) were still at increased risk of postoperative AKI even after adjustment for confounding factors, further implying that low serum bicarbonate concentrations may be independently associated with a decline in renal function after cardiac surgery (Table 5). Similarly, even after propensity score matching, a significant increase in the risk for CSA-AKI development still persisted in group 1 (OR 5.77, 95% CI 2.01–16.55, *P* < 0.001) and group 2 (OR 4.07, 95% CI 1.48–11.18, *P* = 0.01) compared to group 3. (Supplementary Tables 1 and 2.)

**TABLE 5:**
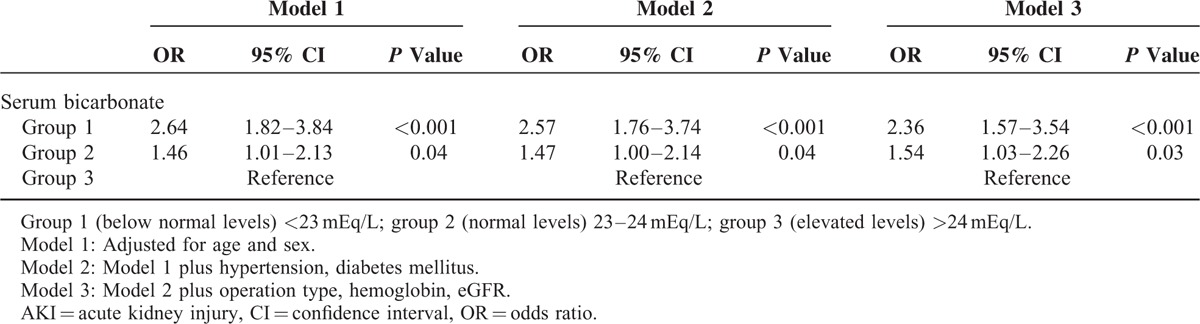
Multivariate Logistic Regression Analysis of Association Between Serum Bicarbonate Level Groups and Cardiac Surgery-Associated AKI Development

## DISCUSSION

Even though acidosis has been considered to be a risk factor for renal function decline in patients with CKD, little is known about acidosis's association with the development of AKI after cardiac surgery. The present study shows that slightly lower than normal serum bicarbonate levels are associated with consecutive diminished kidney function and adverse outcome in patients undergoing cardiac surgery.

AKI is a common complication of cardiac surgery. Previous studies have demonstrated that AKI is accompanied in almost 30% of patients who have undergone cardiac surgery—even though the incidence of CSA-AKI varies somewhat depending on the characteristics of the study population.^6,25–27^ The incidence of AKI in this study was comparable to incidences found in earlier studies. However, the proportion of patients requiring RRT was slightly higher than the proportions in previous investigations,^3,28^ which could be attributed to a relatively higher proportion of valvular surgery cases. Furthermore, recently easier accessibility of continuous RRT might be partly accountable for the increase in the number of patients with AKI who receive RRT after cardiac operations.

CSA-AKI is known to be associated with mortality and morbidities, including postoperative complications and infection.^3,6,7^ In accordance with previous studies, postoperative ICU stay and total hospital admission durations were significantly longer for patients who experienced CSA-AKI. In addition, overall postoperative mortality rates were significantly higher in the CSA-AKI group. Meanwhile, the durations of ICU stay and hospital admission were significantly longer in patients with lower preoperative serum bicarbonate concentrations. Based on those findings, it could be inferred that low serum bicarbonate levels affected the development of CSA-AKI, which might have led to worse clinical outcomes. A recent observational study, too, found that lower serum bicarbonate concentrations predicted the development of AKI and that mechanical ventilation was required more frequently in critically ill patients with AKI,^29^ thereby supporting a close association between the serum bicarbonate levels and renal dysfunctions as well as the long durations of ICU stay and hospital admission that were observed in the present study.

Several risk factors have been previously verified to be closely associated with the development of CSA-AKI. Previous studies showed that a variety of comorbidities such as hypertension, diabetes, and underlying renal insufficiency were implicated in AKI development after cardiac surgery.^2,6,30–33^ Moreover, the types of cardiac operations also were demonstrated to be associated with CSA-AKI. Compared with off-pump CABG, cardiac surgery using cardiopulmonary bypass circuit increases the risk of CSA-AKI,^34,35^ and thus patients receiving cardiac valve surgery have higher incidences of postoperative AKI than do those undergoing off-pump CABG operations.^36^ Consistent with the results of previous studies, we, too, found that hypertension, diabetes, low eGFR, and valvular heart surgery were associated with CSA-AKI. In contrast, patients who received combined valve and CABG operation were not at significantly higher risk of AKI, which may be because of an extremely small number of patients undergoing combined surgeries. Similarly, because CABG was performed by an off-pump method in most of the patients, the difference in the incidence of AKI between CABG patients by cardiopulmonary bypass and by off-pump did not reach statistical significance.

The underlying exact mechanism for the association between low serum bicarbonate concentrations and CSA-AKI is not clear, but several possibilities can be considered on the basis of the results of previous animal experiments. Metabolic acidosis has been shown to induce medullary ammonia production, which in turn activates the alternative complement pathway and aggravates tubular inflammation in an animal model of AKI.^37^ Furthermore, excessive acid loading in mice resulted in activation of the intrarenal rennin–angiotensin–aldosterone system.^14^ In addition, sodium bicarbonate is demonstrated to increase renal medullary oxygen delivery and to reduce renal iron-mediated free-radical formation.^38^ Based on those findings, some investigators attempted to prevent AKI through urine alkalization by sodium bicarbonate infusion, but the results of those trials were inconsistent.^39,40^ The results of the present study could aid in the selection of patients at high risk of AKI, and such therapeutic interventions might be of benefit to them.

There is a possibility that the serum carbon dioxide levels may not represent the true serum bicarbonate concentrations in conditions such as respiratory alkalosis or sepsis. Comorbid conditions of the studied patients were re-examined to investigate the probable consequence of serum carbon dioxide change due to these disorders. None of the patients had active infection including sepsis. In addition, chronic obstructive pulmonary disease and heart failure were accompanied in 5.4% and 19.4% of the patients, respectively, but the prevalence of these comorbidities was comparable among the 3 groups (Supplementary Table 3). Moreover, when additional analysis was done with 191 patients in whom arterial blood gas analysis (ABGA) was done at the same time of serum bicarbonate level test, only 6 patients were found to have respiratory alkalosis (result not shown). Furthermore, the low serum bicarbonate level was still a significant predictor of CSA-AKI even in analyses excluding these patients (Supplementary Table 4). Similarly, even after propensity score matching, a significant increase in the risk for CSA-AKI development still persisted in groups 1 and 2 compared to group 3. Therefore, low serum bicarbonate levels due to low carbon dioxide levels may have little impact on the final results of the study.

There are a number of limitations in this study. First, even though limitation in patient selection is inevitable because of the retrospective design, all patients who underwent cardiac surgery during the given study period were included in the analysis in order to minimize selection bias. In addition, because the patients underwent heart operations in a single center, most of the operation-procedure-related factors as well as postoperative management practice were surmised to be controlled appropriately. Second, ABGA results were not available in all of the patients. Arterial pCO_2_ and pH were not determined, thereby opening the possibility that some of the alteration in serum bicarbonate levels could be a consequence of respiratory problems. Third, the serum bicarbonate levels used in our study were values from a single-time-point. Serum bicarbonate levels could fluctuate overtime. Considering that the mean interval between serum bicarbonate level measurement and operation was about 1 day (mean 24.9 ± 13.2 hours) the possibility of a large level change may not be great. To validate whether serum bicarbonate levels were stable or not during a short time period, 152 patients in whom repeated measurements of serum bicarbonate were done within 96 hours before operation were re-evaluated. In result, we found that there was a significantly close correlation between the mean levels of serum bicarbonate of each patients during the 96 hours and the bicarbonate levels used in the present study (*R* = 0.856, *P* < 0.001). In addition, the serum bicarbonate group was changed during the 96 hours in only 2 patients. Nonetheless, the chance of misgrouping due to bicarbonate value fluctuation could not be completely excluded. Fourth, the number of patients was relatively small. Despite some aforementioned weak points, the results of the study found that serum bicarbonate concentrations were significantly associated with an increase in the risk of CSA-AKI regardless of the etiology of lower serum bicarbonate levels.

In conclusion, even slightly lower than normal serum bicarbonate levels before cardiac operation are associated with the development of postoperative AKI independent of baseline eGFR and other clinical and demographic factors. Further studies are needed to clarify whether strict correction of bicarbonate levels close to normal limits may have a protective role in preventing further AKI development after cardiac surgery.

## Supplementary Material

Supplemental Digital Content
