# Supplementary material for: Preoperative Low Serum Bicarbonate Levels Predict Acute Kidney Injury After Cardiac Surgery
Source: Medicine (Baltimore). 2016 Apr 1;95(13):e3216. doi: 10.1097/MD.0000000000003216 (PMC4998548; doi:10.1097/MD.0000000000003216)
Supplement: Supplemental Digital Content [file medi-95-e3216-s001.pdf]

SUPPLEMENTARY TABLE 1. Baseline characteristics of patients by pre-operative serum bicarbonate level groups after propensity score matching

	Group 1 (n =32)	Group 2 (n=43)	Group 3 (n=66)	*P* value
Age (years)	64.0 ± 12.5	62.7 ± 13.1	63.5 ± 12.6	0.59
Male (%)	18 (56.3)	26 (60.5)	44 (66.7)	0.58
Hypertension (%)	15 (46.9)	18 (41.9)	20 (30.8)	0.22
Diabetes mellitus (%)	10 (31.3)	7 (16.3)	9 (13.6)	0.24
COPD (%)	3 (9.4)	1 (2.3)	3 (4.5)	0.37
Heart failure (%)	7 (21.9)	5 (11.6)	15 (22.7)	0.32
Type of surgery				
Valve surgery (%)	16 (50.0)	22 (51.2)	42 (63.6)	0.30
CABG + Valve surgery (%)	0 (0)	2 (4.7)	3 (4.5)	0.47
CABG (%)	16 (50.0)	19 (44.2)	21 (31.8)	0.17
eGFR (ml/min/1.73m^2)	77.9 ± 21.8	82.3 ± 19.2	86.9 ± 18.9	0.09
Hemoglobin (g/dl)	11.8 ± 2.4	11.4 ± 2.4	11.6 ± 2.1	0.72
Serum glucose (mg/dl)	137.1 ± 59.3	123.0 ± 37.5	119.8 ± 44.6	0.22
Albumin (g/dl)	3.4 ± 0.8	3.3 ± 0.8	3.2 ± 0.8	0.45

Data are expressed as either mean ± standard deviations or numbers (%) except where noted.

Group 1 (below normal levels) <23 mEq/l; group 2 (normal levels) 23–24 mEq/l; group 3 (elevated levels) >24 mEq/l.

CABG = coronary artery bypass graft, COPD = chronic obstructive pulmonary disease, eGFR = estimated glomerular filtration rate

SUPPLEMENTARY TABLE 2. Logistic regression analysis of association between serum bicarbonate level groups and cardiac surgery-associated AKI development after propensity score matching

	OR	95% CI	*P* value
Serum bicarbonate			
Group 1	5.77	2.01-16.55	<0.001
Group 2	4.07	1.48-11.18	0.01
Group 3	Reference		

Group 1 (below normal levels) <23 mEq/l; group 2 (normal levels) 23–24 mEq/l; group 3 (elevated levels) >24 mEq/l.

AKI = acute kidney injury, CI = confidence interval, OR = odds ratio

SUPPLEMENTARY TABLE 3. Prevalence of comorbidities among pre-operative serum bicarbonate level groups

	Group 1 (n =193)	Group 2 (n=226)	Group 3 (n=456)	*P* value
Hypertension (%)	80 (41.5)	72 (31.9)	141 (30.9)	0.03
Diabetes mellitus (%)	40 (20.7)	39 (17.3)	76 (16.7)	0.46
COPD (%)	9 (4.7)	17 (7.5)	21 (4.6)	0.25
Heart failure (%)	39 (20.2)	46 (20.4)	85 (18.6)	0.83

Data are expressed as numbers (%).

Group 1 (below normal levels) <23 mEq/l; group 2 (normal levels) 23–24 mEq/l; group 3 (elevated levels) >24 mEq/l.

COPD = chronic obstructive pulmonary disease

SUPPLEMENTARY TABLE 4. Multivariate logistic regression analysis of association between serum bicarbonate level groups and cardiac surgery-associated AKI development after excluding the patients with respiratory alkalosis

	Model 1			Model 2			Model 3		
	OR	95% CI	*P* Value	OR	95% CI	*P* Value	OR	95% CI	*P* Value
Serum bicarbonate									
Group 1	2.60	1.78–3.78	<0.001	2.55	1.73–3.69	<0.001	2.25	1.50–3.28	<0.001
Group 2	1.46	1.00–2.13	0.05	1.47	1.00–2.14	0.04	1.51	1.02–2.26	0.04
Group 3	Reference			Reference			Reference		

Group 1 (below normal levels) <23 mEq/l; group 2 (normal levels) 23–24 mEq/l; group 3 (elevated levels) >24 mEq/l.

Model 1: adjusted for age and sex

Model 2: model 1 plus hypertension, diabetes mellitus

Model 3: model 2 plus operation type, hemoglobin, eGFR

AKI = acute kidney injury, CI = confidence interval, OR = odds ratio
